# DYNEELAX Robotic Arthrometer Reliability and Feasibility on Healthy and Anterior Cruciate Ligament Injured/Reconstructed Persons

**DOI:** 10.1155/2024/3413466

**Published:** 2024-04-15

**Authors:** Nuno Nascimento, Roula Kotsifaki, Emmanouil Papakostas, Bashir A. Zikria, Khalid Alkhelaifi, Elisabet Hagert, Bruno Olory, Pieter D'Hooghe, Rod Whiteley

**Affiliations:** Aspetar Orthopaedic and Sports Medicine Hospital, Sports City Street, P.O. Box 29222, Doha, Qatar

## Abstract

**Background:**

Anterior cruciate ligament (ACL) injuries are commonly assessed using clinical examination and magnetic resonance imaging, but these methods have limitations in reproducibility and quantification. Instrumented laxity measurements using devices, like the DYNEELAX®, offer an alternative approach. However, to date, there is no human data on the DYNEELAX® and the reliability of these devices remains a subject of debate, and there is no consensus on appropriate knee tightening levels for testing. We hypothesized that the DYNEELAX®, with standardized knee tightening, would provide reliable measurements of knee laxity in adult volunteers.

**Methods:**

This prospective cohort study involved 48 pain-free adult volunteers. Laxity measurements were taken using a robotic-type motorized instrument (DYNEELAX®) on two separate occasions, at least 1 h and no more than 8 h apart, with knee tightening forces of 90 N ± 5 N. Metrics of anterior tibial translation and internal/external tibial axial rotations were recorded.

**Results:**

The device displayed excellent intrarater reliability for all the metrics, with intraclass correlation coefficients ranging from 0.91 to 0.96. Anterior translation exhibited the highest reliability (intraclass correlation coefficient = 0.96), with a minimum detectable change of 0.83 mm.

**Conclusions:**

DYNEELAX® is reliable in measuring knee laxity in adult volunteers when using standardized stabilizing knee tightening forces of 90 ± 5 N. The most sensitive measurement parameters (in terms of minimum detectable change as a proportion of the observed range) were anterior translation (in mm) at 150 N and secondary compliance.

## 1. Introduction

The integrity of the anterior cruciate ligament (ACL) after injury is usually assessed using history, clinical examination, and magnetic resonance imaging (MRI) techniques. A common aspect of the clinical examination is manual laxity testing [1, 2]. Clinical laxity examination techniques are shown to be poorly reproducible and examiner-dependent [3, 4]. Furthermore, the results of these maneuvers are qualitative, which does not enable quantitative comparisons between patients and examiners [5]. Instrumented laxity measurements (“laximetry”) may offer a valid alternative for use in the clinical diagnosis and follow-up of ACL injured and reconstructed patients [6]. Laximetry devices such as the KT-1000, the GNRB®, or the Telos are used to look for side-to-side differences in laxity between two knees of a patient, as well as at different time points in the same patient [7–9]. However, some studies report poor reproducibility and accuracy of these devices [7, 10–15]; the GNRB® seems to show superior results [5, 6, 16–22].

Side-to-side comparisons increase the accuracy of the diagnosis [4, 19] and are an important element of postoperative laxity monitoring after ACL reconstruction [18]. The GNRB® and DYNEELAX® are robotic-type motorized devices, which do not require human application of the exterior applied forces. By mechanically standardizing the forces applied to the knee in terms of magnitude and rate of application, it is thought that better reliability may be achieved in comparison to devices which require human application of force.

While the amount of posteriorly directed stabilizing force on the patella and ankle has been proven to directly affect the laximetry results [6, 14, 17], with higher forces resulting in more reliable tests, most of the existing literature on GNRB® used the manufacturer's knee tightening recommendations of a minimum of 30–50 N [5, 6, 16–22]. To date, there is no consensus on the minimum acceptable knee tightening required for a reliable measurement and there is no objective way to measure ankle tightening, since it is completely dependent on tester's feel and experience [17]. An unpublished pilot investigation in our facility suggests that knee laxity results are higher with knee tightening values inferior to approximately 75 N, after which they appear to stabilize, with the most reliable measurements being achieved from 90 N ± 5 N.

The DYNEELAX®, which is a recent update to the original GNRB® devices, is attached to LDA® couch which allows to standardize the trunk position at 30° to minimize hamstring tension and cocontraction and claims to provide better instrumented measurement of posterior-to-anterior as well as rotational laxity of the tibiofemoral joint. Sensors record the displacement of the tibia along with the associated anteriorly directed force, as well as its internal/external rotation during externally applied torque to the foot and ankle. Results are plotted with translations and rotation curves, which allow for ACL and peripheral structures' compliance to be examined via the displayed force-displacement curves [20, 23]. To our knowledge, only one reliability study has been conducted on this device [17]; however, this study used a prosthetic leg as a model.

As no human data exist regarding the reliability of the DYNEELAX®, the aim of this study is to document the test-retest reliability of the DYNEELAX® in adult volunteers using standardized test procedures which ensure firm proximal stabilization. Our primary hypothesis is that DYNEELAX, when using higher knee tightening forces, is a reliable device for measuring translational and rotational (internal and external) knee laxity.

## 2. Materials and Methods

### 2.1. Patients

This prospective observational cohort study was conducted at the Assessment and Movement Analysis Lab of our institution. Inclusion criteria were as follows: >18 years, pain-free, and no significant knee effusion which prevented testing. A total of 48 participants were included in this study. For each participant, we recorded the following data: knee status (no ACL injury, ACL-injured, and ACL-reconstructed), date of birth, gender, body weight and height, and body mass index (BMI). This study measured a total of 96 knees, out of which 82 were healthy (no previous ACL injury), 11 had undergone reconstruction, and 3 were ACL-injured. One participant was tested only for anterior translation due to reported pain in the ankle during the rotation test. All the participants provided informed consent, and the ethical approval was provided by the Aspire Zone Foundation Institutional Review Board (E202301052).

### 2.2. Knee Laxity Measurements

**Figure 1:**
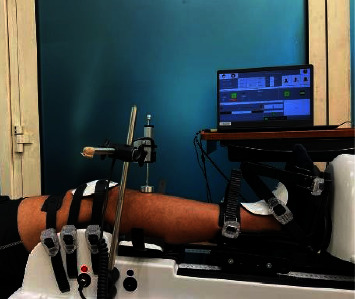
Example of the DYNEELAX® setup.

The DYNEELAX® is an automated device for laxity measurement of anteroposterior tibial translation and internal/external tibial axial rotations. During a clinical measurement, participants were seated with their trunk inclined to 30° relative to the examination table, with the leg placed on a rigid adjustable leg support, which is standardized at 20° of knee flexion, with the knee at neutral internal/external rotation [24], with the inferior pole of the patella facing anteriorly and centered with the knee-cup hole. A displacement transducer records the posterior-to-anterior relative displacement of the anterior tibial tubercle. Ankle tightening was controlled by the operator, and it was as tight as possible, without being painful to the participant. Limb positioning was controlled by using the same limb length as on the previous test and by aligning the inferior pole of the patella with the knee-cup front hole. The device allows a side-to-side comparison to be performed of translational and rotational laxity. The DYNEELAX® measurements were performed by two device-experienced operators, with 18 and 35 years of clinical experience and approximately 3500 DYNEELAX patient tests, on two separate occasions at least one hour (but not more than eight hours) apart. The participant's positioning and setup were similar to the manufacturer's recommendations, except for aiming to apply a proximal stabilizing force of 90 N ± 5 N to the knee. Anterior translation was first assessed, followed by rotation, for all knees. The leg to be tested first was determined by a coin flip. For both anterior translation and internal/external rotation measurements, 3 familiarization repetitions were performed, in order to reduce apprehension and muscle cocontraction (as per manufacturer's recommendations), followed by the test (3 tests at a maximum of 150 N for anterior translation and 3 tests at a maximum of 5 Nm for internal and external rotation). The same conditions were applied for both sessions, with a symmetrical stabilizing pressure applied to each leg, in each test for each participant. The results are plotted on the DYNEELAX® user interface with several curves for translations (mm/N) and rotations (deg/Nm). Each increment of 1N in translations and 0.1 Nm in rotations generates a point on the curves. Compliance metrics are derived from each test by averaging the slopes between all points within specified boundaries. Translation curves are analyzed for primary compliance (PCa) and secondary compliance (SCa), calculated between 30 N and 70 N, and from 100 N up to the maximum force applied (final point), respectively. Conversely, for internal and external rotations, a singular compliance value is computed within the range of 2 Nm to the maximum torque applied (final point) [17]. In addition here we report the anterior translation (in mm) at 150 N.  Figure 1 represents the DYNEELAX® setup.

### 2.3. Statistical Analysis

Statistical analyses were performed using Microsoft Excel® (Office 365, Microsoft, Redmond, CA, USA), JMP (v16.0, SAS), and Python (version 3.9 using the Pingouin 0.5.3 package). Test-retest reliability was documented using intraclass correlation (ICC)_(2,1)_ (absolute agreement) and Bland–Altman plots which allowed estimation of bias and minimum detectable change.

## 3. Results

### 3.1. Characteristics of the Participants

A total of 48 participants (34 males, 71%, and 14 females, 29%) were assessed. Participants' characteristics are presented in Table 1.

### 3.2. Reliability Results

Reliability and other clinimetrics are presented in Table 2. Bland–Altman plots and joint plots for each of the reliability analyses (anterior translation at 150 N; PCa and SCa; IR and ER in degrees at 5 Nm, IR and ER slope) are provided as Supplementary Figures 1to 14 and show no significant mean differences between the two measures.

## 4. Discussion

### 4.1. DYNEELAX® Reliability

Here we have demonstrated that the DYNEELAX® displays excellent intratest reliability results when using 90 N ± 5 N for all the metrics, with ICC ranging from 0.91 to 0.96. Anterior tibial translation was the measurement that displayed the highest reliability (ICC [95% CI] = 0.96 [0.94–0.97] and a minimum detectable change of 0.83 mm). The minimum detectable change (MDC) of 0.83 mm represents 17% of the mean value for this measurement.

Despite the excellent reliability results, it is important to acknowledge the magnitude of the MDC as a percentage of the calculated mean values for each metric to better understand the test-retest variance associated with this device, which ranged from about 17% to about 28%. Any observed deviation equal to or greater than the calculated MDCs, in any of the clinimetrics, should be considered as a substantial difference. As previously noted [14, 17], some sources of error could be variations in patient positioning, reflex muscle contraction, and ankle tightening, which are hard to control as there is no objective manner to measure these variables.

The DYNEELAX® introduced the capability to measure not only anterior tibial translation but also internal and external rotation laxity. To contextualize our findings, we reviewed existing literature on the GNRB®'s reliability and performance, as only one reliability study can be found in the literature on the DYNEELAX®. A few studies, including those by Vauhnik et al. [22] and Mouarbes et al. [14], reported moderate intrarater reliability when using the GNRB® arthrometer. The reliability seemed to vary based on the applied anterior thrust force, knee tightening, and patient's positioning. In contrast, one recent study [25] using GNRB® showed higher ICC values, which might have been attributed to a better control of patellar stabilization, participant positioning, and recording of hamstring activation with EMG. One crucial aspect highlighted in the literature was the sensitivity of the GNRB® to the knee tightening, participant positioning, and soft tissue motion errors [25], impacting tibial rotation errors during the test. This sensitivity could lead to variance between measurements [6, 14, 16]. One thing most previous studies on the GNRB® had in common is that they have used low knee tightening, as recommended by the manufacturer (minimum of 30–50 N), which we believe could have affected their results.

Mouarbes et al. [14] looked at measurement variability with higher knee tightening forces (75 N–90 N and >90 N), in anterior translation measurements, and reported intraclass differences between measurements ≥0.8 mm in 50% of the cohort and ≥1.5 mm for 25% of the cohort when performing side-to-side measurements, with a significant decrease in anterior translation with higher knee stabilizing forces. However, the authors reported low intraclass correlation for the GNRB® device, in contrast to our findings. This observation could potentially be attributed to the distinct nature of the GNRB® device, as well as its lack of integration with an LDA® couch for standardizing the patient's trunk positioning.

Cojean et al. [17] were the only authors who examined the DYNEELAX® reliability, although, using a prosthetic leg, which may not replicate laxity found in a living human. Nevertheless, their study reported excellent reliability results for the DYNEELAX®, which matches our findings with real knees. Cojean et al. [17] also found a high sensitivity to knee tightening, ankle tightening, and patella positioning. Higher variability was noted in rotation measurements, highlighting that rotations may be inherently noisier measurements. This aligns with our findings, where rotations were reliable but exhibited more variability (an MDC of approximately 25% of the range observed) than anterior tibial translation (approximately 17%).

In our study, a knee tightening of 90 N ± 5 N and maximum anterior translation thrust of 150 N were applied. This figure was arrived at after an unpublished pilot investigation conducted in our facility that suggests that knee laxity results are higher with knee tightening values inferior to approximately 75 N, after which they appear to stabilize, with the most reliable measurements being achieved from 90 N ± 5 N. In this pilot study, we have also compared differences in ACL stiffness across the ranges: 100–134 N, 100–150 N, 100–200 N, and 150–200 N. No meaningful differences were found when using a maximum force of 150 or 200 N. Accordingly, with an aim of reducing patient discomfort and possibility of injury, we limited our testing to a maximum of 150 N of anterior force. To date we have not noted a single adverse event when using this approach. This does not, of course, mean that there is no possibility for injury, especially in the presence of ligament laxity, and suitable precautions must always be taken to protect the participant's knee.

### 4.2. Clinical Impact

By using higher stabilizing forces (90 ± 5N) and lower applied anterior translation forces (150 N), excellent reliability is demonstrated for metrics of anterior translation and rotational laxity. The MDC data presented here can be used to infer test-retest change within individuals for the different metrics examined.

### 4.3. Limitations

Some variables that were previously shown to significantly impact laxity measurements (participant's positioning, ankle tightening, and muscle cocontraction) and to be highly dependent on the operator's experience and participant tolerance were not controlled in this study. Additionally, this study included healthy, injured, and reconstructed knees, reflecting patients encountered in clinical practice; however, future research may find differing reliability for these subgroups, although this was out of scope for the current investigation.

## 5. Conclusion

The DYNEELAX® displayed excellent reliability when performed with (high) standardized stabilizing forces (90 N ± 5 N). The most sensitive results (in terms of MDC as a proportion of the observed range) were for anterior translation (in mm) at 150 N and SCa.

## Figures and Tables

**Table 1:** Participants' characteristics.

	Mean ± SD	Range
Age (years)	35.8 ± 10.5	18–56
Height (cm)	175 ± 8.1	160–193
Weight (kg)	78.3 ± 16.4	55–135
BMI (kg/m^2^)	25.4 ± 4.1	18.1–42.1

SD, standard deviation; BMI, body mass index; cm, centimetres; kg, kilograms.

**Table 2:** Mean, standard deviation, ICC, SEM, and MDC results for the test-retest.

	Mean ± SD	ICC (95% CI)	SEM	MDC	MDC (%)
Translation_150_ (mm)	4.9 ± 1.5	0.96 (0.94 to 0.97)	0.30	0.83	16.8
PCa (*μ*m/N)	36.4 ± 15.3	0.95 (0.93 to 0.97)	3.32	9.21	25.3
SCa (*μ*m/N)	30.3 ± 15.3	0.91 (0.87 to 0.94)	2.00	5.53	18.3
IR_5_ (°/5 Nm)	10.9 ± 4.4	0.94 (0.91 to 0.96)	1.08	2.98	27.3
ER_5_ (°/5 Nm)	11.3 ± 4.9	0.96 (0.93 to 0.97)	1.04	2.88	25.5
IR_slope_	2.8 ± 0.9	0.91 (0.87 to 0.94)	0.26	0.72	25.6
ER_slope_	2.8 ± 1.0	0.93 (0.89 to 0.95)	0.28	0.76	27.7

SD, standard deviation; ICC (95% CI), intraclass correlation coefficient with 95% confidence interval; SEM, standard error of measurement; MDC, minimum detectable change; Translation_150_, translation at 150 N; PCa, primary compliance; SCa, secondary compliance; IR_5_, internal rotation at 5 Nm; ER_5_, external rotation at 5 Nm; IR_slope_, internal rotation slope; ER_slope_, external rotation slope.

## Data Availability

Access to data is restricted due to legal and ethical concerns tied to patient privacy rights.

## Supplementary Materials

Supplementary Materials Bland-Altman and Scatter-plots plots are provided in Supplementary Materials: (1) SF1 - 150 Translation Long Bland Altman; (2) SF2 - External Rotation Slope Bland Altman; (3) SF3 - External Rotation Scatter Plot; (4) SF4 - External Rotation Long Bland Altman; (5) SF5 - External Rotation Slope Bland Altman; (6) SF6 - Internal Rotation Slope Bland Altman; (7) SF7 - Internal Rotation Scatter Plot; (8) SF8 - Internal Rotation Long Bland Altman; (9) SF9 - Internal Rotation Slope Bland Altman; (10) SF10 - Pca Long Bland Altman; (11) SF11 - Sca Long Bland Altman; (12) SF12 - Translation Long Bland Altman.
