# Supplementary material for: DYNEELAX Robotic Arthrometer Reliability and Feasibility on Healthy and Anterior Cruciate Ligament Injured/Reconstructed Persons
Source: Transl Sports Med. 2024 Apr 15;2024:3413466. doi: 10.1155/2024/3413466 (PMC11023723; doi:10.1155/2024/3413466)
Supplement: Supplementary Materials — Bland-Altman and Scatter-plots plots are provided in Supplementary Materials: (1) SF1 - 150 Translation Long Bland Altman; (2) SF2 - External Rotation Slope Bland Altman; (3) SF3 - External Rotation Scatter Plot; (4) SF4 - External Rotation Long Bland Altman; (5) SF5 - External Rotation Slope Bland Altman; (6) SF6 - Internal Rotation Slope Bland Altman; (7) SF7 - Internal Rotation Scatter Plot; (8) SF8 - Internal Rotation Long Bland Altman; (9) SF9 - Internal Rotation Slope Bland Altman; (10) SF10 - Pca Long Bland Altman; (11) SF11 - Sca Long Bland Altman; (12) SF12 - Translation Long Bland Altman. [file 3413466.f1.zip › Supplementary Figure 06 IR at 5Nm Jointplot.pdf]

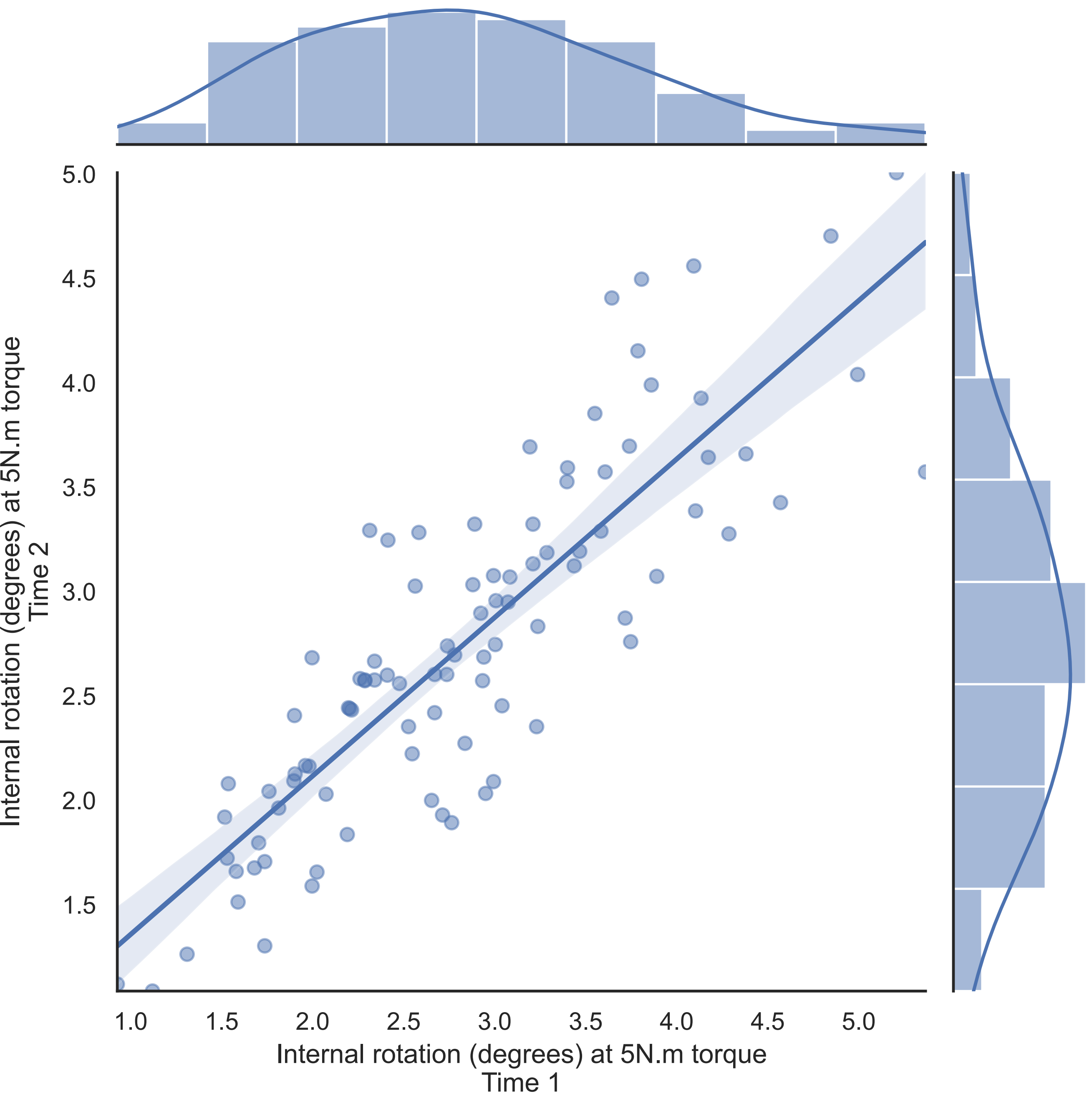

Supplementary Figure 06: Jointplot of tibial internal rotation (degrees) at 5N.m of applied torque at the two time points.
